# Field-Induced Single-Ion Magnet Behavior in Nickel(II) Complexes with Functionalized 2,2′:6′-2″-Terpyridine Derivatives: Preparation and Magneto-Structural Study

**DOI:** 10.3390/molecules28114423

**Published:** 2023-05-29

**Authors:** Francisco Ramón Fortea-Pérez, Julia Vallejo, Teresa F. Mastropietro, Giovanni De Munno, Renato Rabelo, Joan Cano, Miguel Julve

**Affiliations:** 1 Instituto de Ciencia Molecular (ICMol), Departament de Química Inorgànica, Universitat de València, 46980 Paterna, Spain; francisco.fortea@uv.uv (F.R.F.-P.); julia.vallejo@uv.es (J.V.); renato.rabelo@uv.es (R.R.); 2 Dipartimento di Chimica e Tecnologie Chimiche, Università della Calabria, 87036 Rende, Italy; teresafina.mastropietro@unical.it; 3 Instituto de Química, Universidade Federal de Goiás, Goiânia 74690-900, Brazil

**Keywords:** nickel, crystal structure determination, functionalized 2,2′:6′,2″-terpyridine, nitrogen ligands, magnetic properties, theoretical calculations

## Abstract

Two mononuclear nickel(II) complexes of the formula [Ni(terpyCOOH)_2_](ClO_4_)_2_∙4H_2_O (**1**) and [Ni(terpyepy)_2_](ClO_4_)_2_ MeOH (**2**) [terpyCOOH = 4′-carboxyl-2,2′:6′,2″-terpyridine and terpyepy = 4′-[(2-pyridin-4-yl)ethynyl]-2,2′:6′,2″-terpyridine] have been prepared and their structures determined by single-crystal X-ray diffraction. Complexes **1** and **2** are mononuclear compounds, where the nickel(II) ions are six-coordinate by the six nitrogen atoms from two tridentate terpy moieties. The mean values of the equatorial Ni-N bond distances [2.11(1) and 2.12(1) Å for Ni(1) at **1** and **2**, respectively, are somewhat longer than the axial ones [2.008(6) and 2.003(6) Å (**1**)/2.000(1) and 1.999(1) Å (**2**)]. The values of the shortest intermolecular nickel–nickel separation are 9.422(1) (**1**) and 8.901(1) Å (**2**). Variable-temperature (1.9–200 K) direct current (dc) magnetic susceptibility measurements on polycrystalline samples of **1** and **2** reveal a Curie law behavior in the high-temperature range, which corresponds to magnetically isolated spin triplets, the downturn of the χ_M_ *T* product at lower temperatures being due to zero-field splitting effects (*D*). Values of *D* equal to −6.0 (**1**) and −4.7 cm^−1^ (**2**) were obtained through the joint analysis of the magnetic susceptibility data and the field dependence of the magnetization. These results from magnetometry were supported by theoretical calculations. Alternating current (ac) magnetic susceptibility measurements of **1** and **2** in the temperature range 2.0–5.5 K show the occurrence of incipient out-phase signals under applied dc fields, a phenomenon that is characteristic of field-induced Single-Molecule Magnet (SMM) behavior, which herein concerns the 2 mononuclear nickel(II) complexes. This slow relaxation of the magnetization in **1** and **2** has its origin in the axial compression of the octahedral surrounding at their nickel(II) ions that leads to negative values of *D*. A combination of an Orbach and a direct mechanism accounts for the field-dependent relation phenomena in **1** and **2**.

## 1. Introduction

Since the first synthesis of 2,2′:6′,2″-terpyridine (terpy), which dates from more than ninety years ago [1,2], its coordination chemistry has been thoroughly explored, the kinetics and mechanism of formation of its metal complexes, as well as their stability, being also investigated [3,4,5]. Although the three pyridyl rings of this oligopyridine exhibit transoid configurations about the interannular carbon–carbon bonds in the free ligand [6,7,8,9], it preferably adopts a *cis-cis*-configuration acting as a tridentate ligand in the presence of metal ions, and very rare examples of this ligand adopting bidentate or monodentate coordination modes are known [10]. The great stability of its coordination compounds is due to the thermodynamic chelate effect and also to the δ-donor/π-acceptor character of its metal-to-ligand bond. The use of this ligand in the field of supramolecular chemistry and materials science has afforded a plethora of chemical objects, such as racks, ladders and grids [11], helicates [12,13,14], catenanes [15,16,17,18] and dendrimers [19,20,21,22,23,24,25]. Terpy-containing complexes have attracted particular interest also as catalysts [26], some examples being their use in asymmetric catalysis [27], oxidation of alcohols [28,29,30,31,32], cyclopropanation [33], epoxidation [34] and hydrosilylation [35] and as oxygen-binding molecules [36], to name a few. Moreover, their distinct photophysical, electrochemical and magnetic properties are at the origin of their potential use in photovoltaics [37,38,39,40,41,42], light-emitting electrochemical cells [43,44,45,46,47,48], non-linear optics [49,50,51,52,53], spin-crossover-based switching devices [54,55,56,57,58,59,60,61,62,63,64] and medicinal chemistry [65,66,67,68,69,70,71,72].

A great variety of substituents can be introduced into the terpy unit [73], in particular at the 4′ position, to provide not only a means of directionality along the coordination axis, but also to improve its functionality, leading to the possibility of preparing tailor-made, multifunctional homo- and heterometallic assemblies [74,75,76,77,78,79]. In this work, we explore the possibility to cause subtle structural changes at the nickel(II) ion in its bis-terpy compounds by using different substituents at the 4′-position of the terpy ligand that could be responsible for a sizable magnetic anisotropy of this divalent metal ion. Keeping this idea in mind, we prepared and structurally characterized the mononuclear nickel(II) complexes of the formulas [Ni(terpyCOOH)_2_](ClO_4_)_2_∙4H_2_O (**1**) and [Ni(terpyepy)_2_](ClO_4_)_2_∙MeOH (**2**) [terpyCOOH = 4′-carboxyl-2,2′:6′,2″-terpyridine and terpyepy = 4′-[(2-pyridin-4-yl)ethynyl]-2,2′:6′,2″-terpyridine]. Cryomagnetic studies of **1** and **2** were also performed.

## 2. Results

### 2.1. Synthesis, IR Spectroscopy, Thermal Analysis and X-ray Powder Diffraction

The reaction between the nickel(II) perchlorate hexahydrate and the 4′-substituted-terpy derivatives in 1:2 metal-to-ligand molar ratio afforded **1** and **2** as perchlorate salts in good yields. Their chemical identity was confirmed by elemental analyses (C, H, N) and FT-IR spectroscopy [Figures S1(**1**) and S2(**2**) in the Supplementary Materials], and they were further supported by powder X-ray diffraction (PXRD). Indeed, the PXRD patterns of **1** and **2** agree with the calculated ones from the single-crystal X-ray analyses [Figures S3(**1**) and S4(**2**)], confirming the purity of the bulk material.

The occurrence of a broad absorption centered at 3500 cm^−1^ [ν(O-H)] in the infrared spectrum of **1** is indicative of the presence of water molecules involved in hydrogen bonds [80]. Weak intensity peaks in the wavenumber around 3100 cm^−1^ [ν(C-H)] for **1** and **2** support the presence of terpy ligands in these compounds. The medium and strong intensity peaks at 1716 cm^−1^ [ν (C=O)] in the infrared spectrum of **1** are due to the presence of the carboxyl substituent in the terpyCOOH ligand. A weak absorption at ca. 2.280 cm^−1^ [ν (C≡C)] in the infrared spectrum of **2** can be taken as a diagnostic of the presence of the triple carbon–carbon bond of the ethynyl fragment from the terpyepy ligand. The Ni-N bond vibration is generally found at 580–500 cm^−1^. Small shifts towards lower wavenumbers of the C=N and C=C bond vibrations in the region 1550–1450 cm^−1^ for the infrared spectra of **1** and **2** compared to those of the free ligands would suggest their coordination. Finally, the set of strong overlapped peaks centered at 1075 cm^−1^ [ν (Cl-O)] in the infrared spectra of **1** and **2** points out the occurrence of ionic perchlorate groups [81]. All these spectroscopic features were confirmed by the single-crystal X-ray analysis.

Because of the potential explosive character of the perchlorate salts containing organic ligands, the thermogravimetric study of **1** and **2** was limited to the temperature range 25–200 °C (Figure S5 in the Supplementary Materials). Mass losses attributed to 4 water molecules of crystallization in **1** (obsd 8.10%; calcd. 8.14%) and to 1 uncoordinated methanol molecule in **2** (obsd 3.14%; calcd. 3.34%s) start at 32 °C for both compounds, and they are practically completed at ca. 100 °C.

### 2.2. Description of the Crystal Structures of ***1*** and ***2***

The crystal structures of **1** and **2** were determined by single-crystal X-ray diffraction, as provided below. Compound **1** crystallizes in a monoclinic *P*2_1_/*c* space group, while **2** crystallizes in the triclinic crystal system in the *P*(-1) space group. Crystal data and details of the data collection and refinement for **1** and **2** are listed in Table 1. Selected bond lengths and angles are given in Table 2 (**1**) and Table 3 (**2**).

**Figure 1:**
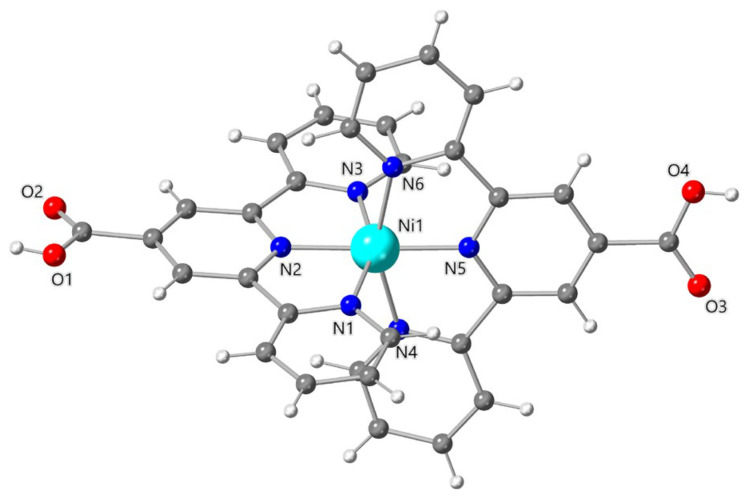
Perspective drawing of the cationic unit of **1** of formula [Ni(terpyCOOH)_2_]^2+^ showing the atom numbering of the nickel(II) surrounding.

The structure of **1** consists of cationic mononuclear bis(2,2′:6′,2″-terpyridine-4′-carboxylic acid)nickel(II) complex cations, perchlorate anions and water molecules of crystallization. The nickel environment in **1** is well described by a slightly compressed octahedral surrounding NiN_6_, the equatorial plane being defined by the N(1)N(3)N(4)N(6) set of atoms, with N(2) and N(5) in axial positions (Figure 1). The mean value of the equatorial Ni-N bond distances from the terpyCOOH ligand [2.112(6) Å] is slightly longer than the axial ones [2.008(6) and 2.003(6) Å for Ni(1)-N(2) and Ni(1)-N(5), respectively] and in agreement with those found for other similar complexes of formulas [Ni(terpyCOO)_2_]∙4H_2_O and Ni(terpyphCOO)_2_]∙5H_2_O [teryphCOOH = 4′-(4-carboxyphenyl)-2,2′:6′,2″-terpyridine], which were previously reported [75,76]. The distortion from the ideal octahedral geometry is mainly due to the reduced bite angles of the tridentate terpy units [values in the range 77.7(3)–78.2(3)°]. The value of the axial N(2)-Ni(1)-N(5) bond angle [178.7(3)°] is very close to that of an ideal octahedron (180°). The two terpyCOOH moieties are not exactly oriented perpendicular to each other, the values of the dihedral angle between the mean planes of the central pyridine rings being 83(1)°. The value of the shortest intermolecular nickel^…^nickel separation is 9.422(1) Å [Ni(1)^…^Ni(1b); symmetry code: (b) = 1 + *x*, *y*, *z*].

The cationic [Ni(terpyCOOH)_2_]^2+^ entities establish hydrogen bonds with the lattice water molecules through the carbonyl-oxygen atoms from the carboxylic acid groups, leading to six-membered rings, as shown in Figure S6 [O^…^Ow = 2.61(1)–2.80(1) Å (see Table S1 in the Supplementary Materials for further details)]. These bonds are responsible for the propagation of the motif along the crystallographic *c* axis. (Figure S7a). The two terpyCOOH ligands play different roles in the crystal packing. Both lateral N(1) and N(3) pyridyl rings of 1 ligand are involved in π-π interactions, which propagate along the crystallographic *a* axis [N(1)/N(3b), N(3)/N(1c); (b) = 1 + *x*, *y*, *z* and (c) = −1 + *x*, *y*, *z*], with an interplanar distance, a dihedral angle and a centroid-centroid distance of 3.717 Å, 2.7° and 4.252 Å, respectively (Figure S7). On the contrary, only the lateral N(6) ring and the carboxylic CO(2)C(32)O(3) group of the second terpyCOOH ligand are involved in stacking-like interactions, with a N(6)/C(32e) dihedral angle of 6.1° [symmetry code: (e) = 1 − *x*, −*y*, −*z*], and an interplanar separation and a centroid-centroid distance of 3.316 and 3.354 Å, respectively (Figure S8).

Interestingly, the first terpyCOOH ligand is almost planar, whereas the second one is slightly bent, as reflected by the values of the dihedral angles between the 2 outer pyridyl rings (2.7° for the first ligand and 13.0° for the second one). Taking into account the π-π interactions occurring between the N(1)/N(3) pyridyl rings of the first terpyCOOH ligand, along with the hydrogen bonds described above, a planar motif is formed in the crystallographic *ac* plane (Figure S7a), whereas pyridyl/pyridyl and pyridyl/COOH interactions lead to a supramolecular ribbon-like motif along the crystallographic *a* axis (Figure S8a).

Additional hydrogen bonds involve the water molecules of crystallization and the perchlorate anions [O(4w)^…^O(1pf) = 2.925 Å; (f) = 2 − *x*, −0.5 + *y*, 0.5 − *z*], and they are responsible for the resulting supramolecular 3D arrangement in **1** (Figure S9).

**Figure 2:**
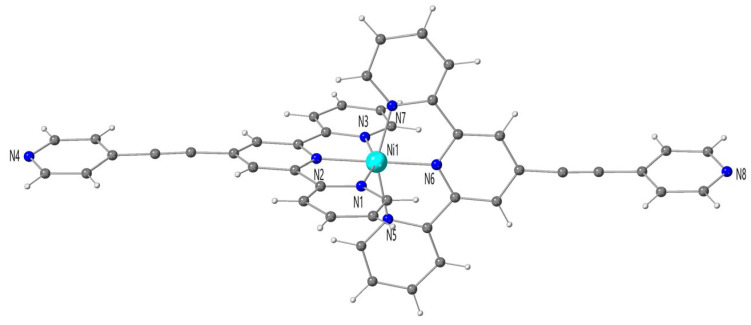
Perspective drawing of the [Ni(1)(terpyepy)_2_]^2+^ complex cation of **2**.

The crystal structure of **2** is made up of a cationic mononuclear bis(2,2′:6′,2″-terpyridine-4′-carboxylic acid)nickel(II) complex cations, two perchlorate anions and one uncoordinated methanol molecule. The nickel environment in **2** is very similar to that in **1**. It is well described by a slightly compressed octahedral surrounding, NiN_6_, the equatorial plane being defined by the N(1)N(3)N(5)N(7) set of atoms, with N(2) and N(6) in axial positions (Figure 2). The mean value of the equatorial Ni-N bond distances from the terpyCOOH ligand [2.123(1) Å] is slightly longer than the axial ones [2.000(1) and 1.999(1) Å for Ni(1)-N(2) and Ni(1)-N(6), respectively]. The shortest Ni-N bond lengths are those related to the inner terpy nitrogen atoms residing in *para* positions to the -COOH (N(2)/N(5)) and epy (N(2)/N(6) groups in **1** and **2**, respectively, as expected looking at similar structurally characterized [Ni(terpy)_2_]^2+^ entities. As in **1**, the distortion from the ideal octahedral geometry is mainly due to the reduced bite angles of the tridentate terpy moieties [values in the range 77(3)–78.0(3)°], and the value of the axial N(2)-Ni(1)-N(6) bond angle [179.2(1)°] is quasi identical to that of an ideal octahedron (180°).

The Ni-terpyepy complex cations are connected by π-π interactions, in which the epy groups and the outer pyridyl rings of only one terpyepy ligand are involved. The values of the separation between the mean planes of the rings containing N(3) and N(4a) and N(4) and N(3a) [symmetry code: (a) = 1 − *x*, −*y*, −*z*] or N(1) and N(8b) and N(8) and N(1b) [(b) = 1 − *x*, 1 − *y*, 1 − *z*)] are 3.344 and 3.484 Å, respectively. The distances between their centroids are 3.680 and 3.846 Å. These planes are almost coplanar, with dihedral angles of 6.2 and 14.3°. Such interactions propagate along the *bc* diagonal, creating a supramolecular 1D motif (Figure S10a). Additional stacking interactions involve the epy pyridyl rings containing the N(4) and N(8) atoms along the crystallographic *c* axis (Figure S10b). They are responsible for the resulting supramolecular 2D arrangement (Figure S11), the mean distance between the N(4) and N(8*c*) and N(8) and N(4*d*) planes being 3.454 Å[(c) = *x*, *y*, −1+*z*; (d) = *x*, *y*, 1+*z*)], with a dihedral angle of 6.0°, and a distance between centroids of 4.176 Å. The supramolecular 3D arrangement is reached by means of π-π stacking occurring between the rings containing the N(1) and N(3e), and N(3) and N(1f) atoms along the crystallographic *a* axis [(e) = −1 + *x*, *y*, *z*; (f) = 1 + *x*, *y*, *z* )] (Figure S12), with a mean separation between the planes, a dihedral angle and a distance between the centroids of 3.377 Å, 2.5° and 3.690 Å, respectively. The 2 terpy moieties of both ligands are almost planar, with dihedral angles of 2.3, 3.7, 5.6 and 4.3° between the N(2)/N(1) and N(2)/N(3), N(6)/N(5) and N(6)/N(7) pairs of rings, respectively. As a substantial difference, the ring of the epy fragment is almost coplanar with the terpy in one of the ligands, the dihedral angle between the N(2)/N(4) rings being 6.9°, while it forms an angle of 80.5° with the ring containing the N(6) atom in the other one. Methanol solvent molecules are arranged in small channels formed along the crystallographic *a* axis (Figure S13). Hydrogen bonding interactions are established between the methanol molecule and the N(4) atom of the epy pyridyl ring. The value of the shortest intermolecular nickel^…^nickel separation in **2** is 8.901(1) Å.

### 2.3. Static (dc) Magnetic Properties of ***1*** and ***2***

**Figure 3:**
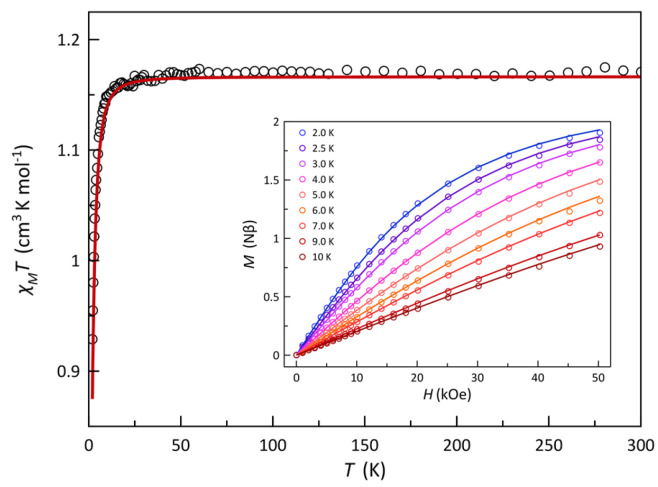
χ_M_*T* against *T* plot for **2**. The inset shows the field dependence of the magnetization at the indicated temperatures. The open circles are the experimental data, and the solid lines correspond to the best−fit curves to the experimental data using the parameters reported in the text, which are obtained through the zfs splitting approach (see text).

The direct current (dc) magnetic properties of **1** and **2** in the form of χ_M_*T* against *T* plots [χ_M_ is the magnetic susceptibility per one Ni^II^ ion] are shown in Figure S14 and Figure 3, respectively. The values of χ_M_*T* at room temperature are 1.19 (**1**) and 1.17 cm^3^ mol^−1^ K (**2**) [μ_eff_ = 3.09 (**1**) and 3.06 BM (**2**)]. They are as expected for one magnetically isolated spin triplet (χ_M_*T* = 1.21 cm^3^ mol^−1^ K with *S*_Ni_ = 1 and *g*_Ni_ = 2.20; μ_eff_ = 3.11 BM). Upon cooling, these values remain constant until ca. 35 K, and they further decrease to 0.84 (**1**) and 0.93 cm^3^ mol^−1^ K (**2**). This downturn of χ_M_*T* at low temperatures for the two compounds could be attributed to weak intermolecular antiferromagnetic interactions and/or zero-field splitting (zfs) effects. Because of the great value of the shortest intermolecular metal–metal separation [ca. 9.4 (**1**) and 8.9 Å (**2**)], the intermolecular magnetic interactions between the local spin triplets can be ruled out. Then, the decrease of χ_M_*T* at low temperatures for **1** and **2** is the fingerprint of the zfs. In this respect, the quasi-saturation values of the magnetization (*M*) at 5 T and 3.0 K for **1** and **2** [ca. 1.77 (**1**) and 1.78 µ_B_ (**2**)], which are somewhat below the expected value (*M*_S_ = *g*_Ni_*S*_Ni_ = 2.20 µ_B_ with *S*_Ni_ = 1 and *g*_Ni_ = 2.20), also support the presence of significant zfs effects [see insets of Figure S14(**1**) and Figure 3(**2**)].

Taking into account the aforementioned features, the magnetic data of **1** and **2** were analyzed through the spin Hamiltonian of Equation (1):

(1) $$\hat{H}=D\left[{\hat{S}}_{z}^{2}-S\left(S+1\right)/3\right]+g\beta H\hat{S}$$

The first and second terms in this expression describe the axial zfs and the Zeeman interaction. In the fitting process, we have neglected the rhombic components of the zfs and considered an average Landé factor (*g*_||_ = *g*_⊥_ = *g*) to avoid overparameterization. The simultaneous analysis of the magnetization data under different applied dc fields and temperatures and the variable-temperature magnetic susceptibility data of **1** and **2** by employing full-matrix diagonalization through the above Hamiltonian, as implemented in the PHI program [82], led to the following set of best-fit parameters: *D* = −6.0 cm^−1^, *g* = 2.17 with *F* = 3.4 × 10^−7^ for **1** and *D* = −4.7 cm^−1^, *g* = 2.16 with *F* = 1.2 × 10^−6^ for **2** (*F* is the agreement factor defined as ∑[*P*_exp_ − *P*_calcd_]^2^/∑[*P*_exp_]^2^, where *P* is the physical property under study). The calculated curves reproduce well the experimental data, as seen in Figure S14 and Figure 3. It deserves to be noted that *D* values ranging from −15.4 to +10 cm^−1^ were reported for octahedral or pseudo-octahedral nickel(II) complexes [83,84,85,86]. In the present examples, the sign and moderate magnitude of the *D* values in **1** and **2** semi-quantitatively agree with those reported previously for homoleptic complexes, with the NiN_6_ chromophore (monodentate nitrogen donors as ligands) exhibiting axial compression [87,88]. In fact, from the empirical expression *D*_mag_ ~ 2{25.8[1−exp(–0.014*D*_str_)]}, which correlates the zfs with structural parameters [88], **1** and **2** should exhibit *D* values of −8.3 and −10.0 cm^−1^, respectively. These values are overestimated, most likely due to the short bite angles at each tridentate derivative in **1** and **2**. Anyway, the sign and trend of the magnitude of *D* in **1** and **2** agree with those obtained from the theoretical study (see below).

### 2.4. Theoretical Calculations on ***1*** and ***2***

To further confirm the validity of the experimental results from magnetometry of **1** and **2**, we carried out theoretical CASSCF/NEVPT2 calculations. They unambiguously support negative values for *D* [−4.8 (**1**) and −5.4 cm^−1^ (**2**) with values of the Landé factor (*g*_iso_) of 2.185 (**1**) and 2.189 (**2**)]. These values agree with those obtained from the analysis of the magnetic data. The theoretical calculations also showed a weak rhombicity in the *zfs* tensor [*E*/*D* = 0.070 (**1**) and 0.057 cm^−1^ (**2**)] due to the D_4h_ pseudosymmetry of the coordination sphere in **1** and **2**, which is the expected one for the bis-terpyridyl metal complexes. However, the impossibility of reaching an ideal equatorial plane for the octahedron at the metal environment achieving usual axial Ni–N bond lengths from the two terpy derivatives prevents a null value for the *E/D* ratio. To fully understand the emergence of the *zfs* and an eas*y*-axis magnetic anisotropy, it is crucial to examine the calculated inputs to the axial contribution of *D*. The results and corresponding energies of the states responsible for each input are summarized in Table S2 (see Supplementary Materials).

A high-spin Ni^II^ ion in octahedral symmetry exhibits a ^3^F ground term, which is split by the ligand field into a ^3^A_2_ ground state, {t_2g_^6^e_g_^2^}, with null orbital momentum (*L*), and two ^3^T_2_ and ^3^T_1_ excited states, {t_2g_^5^e_g_^3^}, which are orbital triplets. Under geometric or electronic distortions of the coordination sphere, these excited states can be further split into up to three new states for each one. For example, an axial distortion can split the ^3^T_2_ state into the ^3^A_1_ and ^3^E states, while a non-regular equatorial plane can lead to the appearance of the ^3^A_1_, ^3^B_1_ and ^3^B_2_ states.

A zero-field splitting (*zfs*), which is mainly characterized by the axial parameter *D*, arises from the interaction of the *m_s_* functions of these excited states with those of the ground ^3^A_1_ one. The contributions of the ^3^B_1_(T_2_) and ^3^B_2_(T_2_) states to *D* are positive, while that of the ^3^A_1_(T_2_) state is negative and of double magnitude. Therefore, these states have the same energy in the O_h_ symmetry, and their contributions cancel each other, resulting in a null *D*. However, a lower symmetry occurs under an axial distortion (*D_4h_* or *C_4v_*,) leading to a non-zero value of *D*. The sign of *D* will depend on the relative stability of the ^3^A_1_(T_2_) and ^3^E(T_2_) states, being negative for the axial compression and positive for the axial elongation.

Under a tetragonal distortion, the ^3^E(T_2_) state is split into ^3^B_1_(T_2_) and ^3^B_2_(T_2_), leading to a rhombicity in the tensor *zfs*, *E*/*D* ≠ 0. The contributions from the ^3^T_12_(^3^F) or other ^1^T_2_(^1^D), ^1^E(^1^D) and ^1^A_1_(^1^G) excited states are usually negligible since they are energetically less stable and distant, except for the two latter ones when strong ligand fields were involved.

The results listed in Table S2 are consistent with the previous comments and also with the following observations:

(i) The primary source of magnetic anisotropy is a second-order spin-orbit coupling, with a negligible contribution from spin–spin interactions.

(ii) The singlet (*D*_S_) and excited states originating from a ^3^T_1_ state (DT31) make insignificant contributions compared to the first three excited triplet states resulting from the low-molecular symmetry-induced split of the ^3^T_2_ state (DT32).

(iii) The ^3^A_1_ state contributes negatively to *D*, while the ^3^B_1_ and ^3^B_2_ states do it positively.

(iv) Due to the low symmetry, there is no cancellation of contributions to *D*. Moreover, the negative sign from the nearest excited state (^3^A_1_) dominates under axial compression in the octahedral coordination sphere.

(v) Although the planarity of the terpy-derived ligands enforces regularity at the metal ion equatorial plane, it is impossible to achieve reasonable Ni–N bond lengths simultaneously for the inner and outer terpyridyl and nitrogen atoms, resulting in a partial distortion that causes a weak to moderate magnetic rhombicity.

(vi) The experimental results and literature data suggest a value of the *g*-factor greater than the one for the free electron, according to the theoretical prediction (2.185 and 2.189 for **1** and **2**, respectively).

As anticipated, the axial compression of the coordination octahedron in **1** and **2** results in the alignment of the *z*-axis of the *zfs* tensor along the vector connecting the central nitrogen atoms of the two terpyridyl fragments coordinated to the Ni^II^ ion (Figure S15).

### 2.5. Dynamic (ac) Magnetic Properties of ***1*** and ***2***

**Figure 4:**
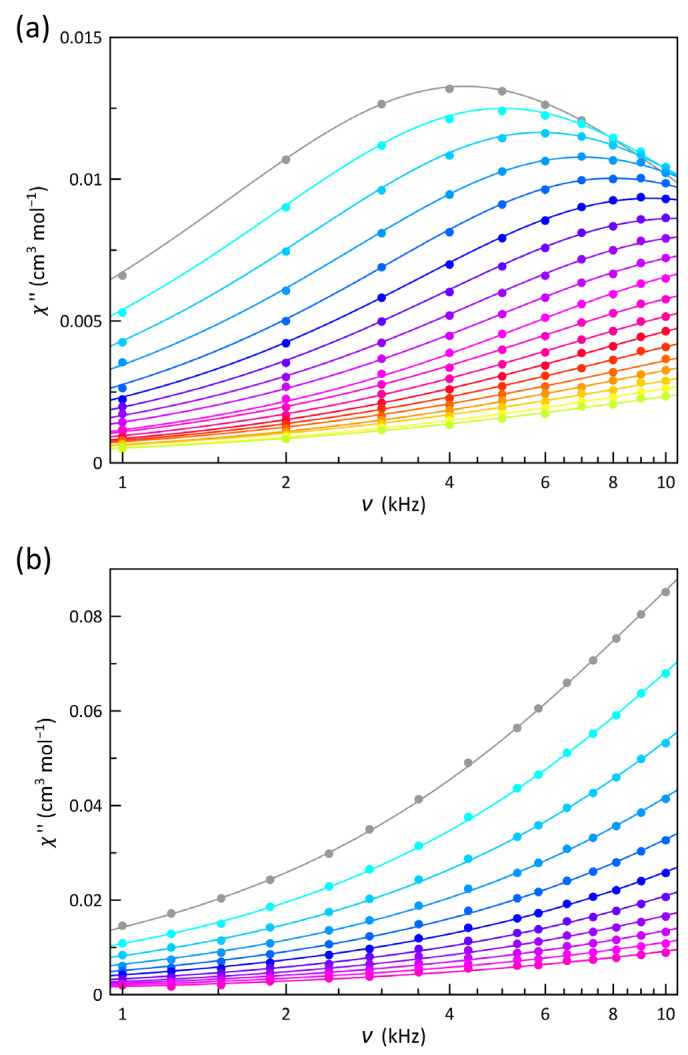
Frequency dependence of χM″ for **1** (**a**) and **2** (**b**) under static magnetic fields of 2.0 and 5.0 kOe, respectively, at ± 5 Oe oscillating magnetic field in the frequency range 10–1.0 kHz (from gray to warmer colors). The solid lines are the best−fit curves obtained through the generalized Debye model.

The ac magnetic properties of **1** and **2** in the form of *χ*_M_′ and *χ*_M_″ vs. *T* or *ν* plots are shown in Figure 4, Figures S16 and S17 (*χ*_M_′ and *χ*_M_″ being the in-phase and out-of-phase ac molar magnetic susceptibilities, whereas *ν* is the frequency of the oscillating ac field in Hz). In the absence of an applied dc magnetic field (*H*_dc_), neither a frequency dependence of *χ*_M_′ nor a *χ*_M_″ signal were observed for **1** and **2**. These features are likely due to a fast quantum tunneling of magnetization (QTM), which becomes less efficient when the external magnetic field is increased, making it then possible for the slow relaxation of the magnetization. Accordingly, frequency-dependent *χ*_M_′ and *χ*_M_″ maxima were observed for **1** when a relatively small dc magnetic field was applied (*H*_dc_ = 2.0 kOe). These maxima shifted toward lower temperatures by decreasing *ν*. In contrast, only very incipient signals occurred for **2** under the same applied *H*_dc_ field. However, by increasing *H*_dc_ to 5.0 kOe, a stronger frequency dependence of *χ*_M_′ and *χ*_M_″ is achieved for **2**. This scenario characterizes the typical field-induced single-molecule magnet (SMM) behavior observed in mononuclear complexes.

**Figure 5:**
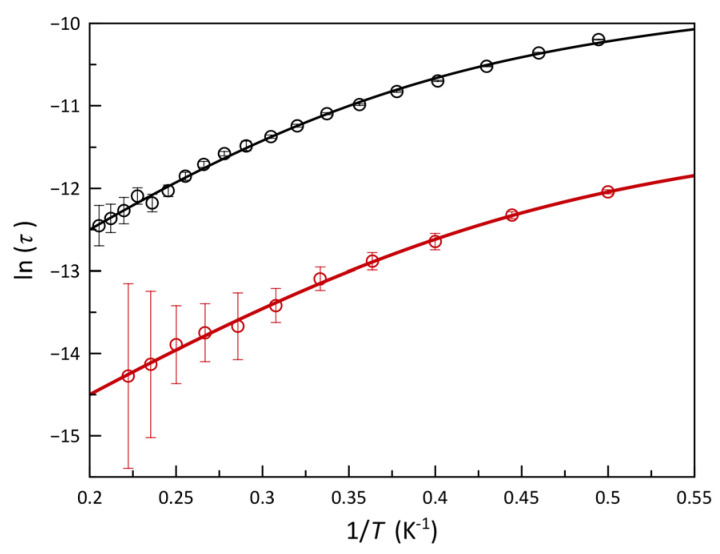
Arrhenius plots for the calculated magnetic relaxation times (τ) of **1** (black) and **2** (red) under applied dc magnetic fields of 2.0 and 5.0 kOe, respectively. Solid lines are the best−fit curves for a thermally activated Orbach plus direct relaxation mechanism described by the equation *τ*^−1^ = *τ*_0_^−1^ exp(−*E*_a_/*k*_B_*T*) + *AT*. Vertical error bars denote the standard deviations.

According to Equations (2) and (3) (*χ*_S_, *χ*_T_, *α* and ω being adiabatic and isothermal magnetic susceptibilities, the exponential factor and 2πν, respectively), the joint analysis of the *χ*_M_′ and *χ*_M_″ vs. *ν* data through the generalized Debye model [89] provided the relaxation time (*τ*), whose temperature dependence is illustrated as an Arrhenius plot in Figure 5. The small values of *α* indicate that all the cobalt(II) ions behave quasi identically; the distribution of the relaxation processes is not broad, even at temperatures around 6.0 K, where that parameter reaches the highest values, but being always far from those corresponding to a spin-glass behavior.

(2) $$\chi_{M}^{\prime }{}_{i}=\chi_{S}+\left(\chi_{T}-\chi_{S}\right)\frac{1+{\left(\omega \tau \right)}^{1-\alpha }Sin\left(\frac{\alpha \pi }{2}\right)}{1+2{\left(\omega \tau \right)}^{1-\alpha }Sin\left(\frac{\alpha \pi }{2}\right)+{\left(\omega \tau \right)}^{2\left(1-\alpha \right)}}$$

(3) $$\chi_{M}^{\prime \prime }{}_{i}=\left(\chi_{T}-\chi_{S}\right)\frac{{\left(\omega \tau \right)}^{1-\alpha }Cos\left(\frac{\alpha \pi }{2}\right)}{1+2{\left(\omega \tau \right)}^{1-\alpha }Cos\left(\frac{\alpha \pi }{2}\right)+{\left(\omega \tau \right)}^{2\left(1-\alpha \right)}}$$

A uniaxial (*D* < 0) and moderate *zfs* to generate a magnetic energy barrier that is low enough to be overcome by the temperature, but high enough to slow down this step, constitutes the ideal scenario for a slow magnetic relaxation arising in spin reversal governed by a thermally assisted Orbach mechanism, $\tau^{-1}=\tau_{0}^{-1}\cdot exp\left(-E_{a}/k_{B}T\right)$ [*τ*_0_, *E_a_*, and *k*_B_ being the pre-exponential factor, the effective energy barrier and the Boltzman constant, respectively]. Two relaxation mechanisms, one of them predominating above 3.0 K (HT) and the other one at lower temperatures (LT), are clearly distinguished in Figure 5. The analysis of these data for **1** with a model of two competing Orbach mechanisms [$\tau^{-1}=\tau_{0,HT}^{-1}\cdot exp\left(-E_{a,HT}/k_{B}T\right)+\tau_{0,LT}^{-1}\cdot exp\left(-E_{a,LT}/k_{B}T\right)$] leads to energy barriers of 12.5(20) (HT) and 3.0(7) cm^−1^ (LT). The first value agrees well with what is expected from the *zfs* parameters obtained by magnetometry (*E_a_* = 2|*D*| = 12.0 cm^−1^) or from the theoretical study (9.7 cm^−1^). However, *E_a_* in the region close to 2.0 K is too small to allow it a physical meaning. Most likely, due to the low thermal energy in this region, the HT Orbach process becomes extremely slow, and other mechanisms, such as a phono-assisted direct one ($\tau^{-1}=A\cdot T$, *A* being a polynomial factor for direct relaxation), eventually prevail. Therefore, an analysis considering the presence of an Orbach relaxation and a direct one [$\tau^{-1}=\tau_{0}^{-1}\cdot exp\left(-E_{a}/k_{B}T\right)+A\cdot T$] is considered more appropriate. This analysis led to the following values: *E_a_* = 9.8(5) cm^−1^, *τ_0_* = 2.9(4) × 10^−7^ s, *A* = 12,200(700) s^−1^K^−1^, and *F* = 1.2 × 10^−5^.

In **2**, the previous situation is repeated. In addition, the scarcity of data at low temperatures makes it difficult to find accurate values of the mechanism operating below 3.0 K. However, better results are found by combining an Orbach and one direct mechanism: *E_a_* = 8.6(6) cm^−1^, *τ_0_* = 5.1(4) × 10^−8^ s, *A* = 65,000(7000) s^−1^ K^−1^ and *F* = 8.7 × 10^−6^. The value of *E_a_* for **2** still agrees with the energy barrier provided by a uniaxial *zfs* (9.4 cm^−1^), and it is smaller than that in **1** because the *zfs* in **2** is also less (|*D*| = 4.7 cm^−1^).

## 3. Materials and Methods

### 3.1. Reagents

Nickel(II) perchlorate hexahydrate, terpyCOOH and the organic solvents were purchased from commercial sources, and they were used as received without any further purification. Terpyepy was prepared as previously described [90], and its purification was performed by liquid chromatography using hexane/dichloromethane (80:20 *v*/*v*) as eluent. 

**Caution!** Perchlorate salts containing organic ligands are potentially explosive and heating has to be avoided. We worked at the mmol scale, the syntheses were carried out in solution and the crystals were obtained by slow evaporation (**1**) or slow diffusion of solvents (**2**) under ambient conditions.

### 3.2. Preparation of the Complexes 

#### 3.2.1. [Ni(terpyCOOH)_2_](ClO_4_)_2_∙4H_2_O (**1**)

A methanolic solution (10 mL) of nickel(II) perchlorate hexahydrate (0.33 g, 0.9 mmol) was added dropwise to a methanolic suspension (5 mL) of terpyCOOH (0.50 g, 1.8 mmol) at 40 °C under stirring. The resulting brownish solution was filtered to remove any small particles, and it was allowed to evaporate slowly in a hood. X-ray quality brownish needles were grown after a few days. They were collected by filtration and dried on filter paper. The yield was 93%. Anal. Calcd. for C_32_H_30_Cl_2_N_6_NiO_16_ (**1**): C, 43.48; H, 3.39; N, 9.50%. Found: C, 43.19; H, 3.31; N, 9.43%. 1:2 Ni:Cl molar ratio. IR (KBr, cm^−1^): 3535 m, br [ν(O-H)], 3105 w [ν(C-H)], 1716 m and 1358 m, [ν(C=O)], 1640 m [ν(C=N], 1535 m, [ν(C=C], 1075 s and 620 s [ν(Cl-O)].

#### 3.2.2. [Ni(terpyepy)_2_](ClO_4_)_2_∙MeOH (**2**)

A dichloromethane solution (3 mL) of terpyepy (0.067 g, 0.2 mmol) was placed at the bottom of a test tube. Then, a methanolic solution (3 mL) of nickel(II) perchlorate hexahydrate (0.037 g, 0.1 mmol) was carefully layered on the top of the previous solution, and the test tube was covered with parafilm and allowed to diffuse at room temperature. Polyhedral maroon crystals were grown after a few days. They were collected by filtration and allowed to dry under ambient conditions. The yield was 75%. Anal. Calcd. for C_45_H_32_Cl_2_N_8_NiO_9_ (**2**): C, 56.41; H, 3.34; N, 11.69%. Found: C, 56,15; H, 3.26; N, 11.57%. 1:2 Ni:Cl molar ratio. IR (KBr, cm^−1^): 3450 br [ν(O-H)], 3108 w [ν(C-H)], 2280 w [ν (C≡C)], 1647 m [ν(C=N], 1560 m [ν(C=C)], 1075 s and 618 m [ν(Cl-O)].

### 3.3. Physical Techniques

Elemental analyses (C, H, N) were carried out by the Servei Central de Support a la Investigació Experimental de la Universitat de València (SCSIE). The value of the Ni-to-Cl molar ratio was determined by X-ray microanalysis with a Philips XL-30 scanning electron microscopy (SEM). FT-IR spectra were recorded on a Nicolet-5700 spectrophotometer as KBr pellets. Powder X-ray diffraction (XPRD) patterns of powdered crystalline samples were collected at room temperature on a D8 Avance A25 Bruker diffractometer by using graphite-monochromated Cu-Kαradiation (α = 1.54056 Å). Thermogravimetric analyses (TG) were performed on crystalline samples of **1** and **2** in the temperature range 25–200 °C with a Mettler Toledo TGA/STDA 851e thermobalance, using alumina crucibles and around 4 mg of each sample. The operating conditions were a dinitrogen flow of 100 mL min^−1^ with a heating rate of 10 °C min^−1^. Direct current (dc) magnetic susceptibility measurements in the temperature range 1.9–300 K under applied dc fields of 5000 G (*T* ≥ 30 K) and 250 G (*T* < 30 K) and variable field (0–5 T) magnetization measurements on crushed crystals of **1** and **2** were performed with a Quantum Design SQUID magnetometer. Variable-temperature (2.0–5.2 K) alternating current (ac) magnetic susceptibility measurements under different applied magnetic fields in the range 0–5 kOe were performed for **1** and **2** by using a Quantum Design Physical Property Measurement System (PPMS). The magnetic susceptibility data of both compounds were corrected for the diamagnetic contributions of the constituent atoms and the sample holder (a plastic bag).

### 3.4. Crystallographic Data Collection and Refinement

X-ray diffraction data on single crystals of **1** and **2** were collected on Bruker-Nonius X8APEXII (**1**) and Bruker D8 Venture (**2**) diffractometers, using graphite-monochromated Mo-Kα radiation (α = 0.71073 Å) at 296(2) (**1**) and 150 (**2**) K (**2**). All calculations for data reduction, structure solution and refinement for **1** and **2** were performed through the SAINT and SADABS programs [91,92]. The structures of **1** and **2** were solved by direct methods and subsequently completed by Fourier recycling using the SHELXTL software package [93,94], then refined by the full-matrix least-squares refinements based on *F*^2^ with all observed reflections. All non-hydrogen atoms were refined anisotropically. One of the perchlorate counterions and the methanol solvent molecules in **2** was found to be involved in somewhat standard disorders. The chlorine and two oxygen atoms of the perchlorate ion and the carbon and oxygen atoms of the solvent were modelled over two sites. The hydrogen atoms of the terpyCOOH and terpyepy ligands in **1** and **2** were set in calculated positions and refined as riding atoms, whereas those of the water molecules in **1** and the methanol solvent molecule in **2** were neither found nor calculated. The final geometrical calculations and the graphical manipulations for **1** and **2** were carried out by using the XP utility within the SHELX [95] and the CrystalMaker program [96]. Crystallographic data have been deposited at the Cambridge Crystallographic Data Centre with CCDC reference numbers 2,252,170 (**1**) and 2,252,171 (**2**).

### 3.5. Computational Details

Calculations based on the second-order N-electron valence state perturbation theory (CASSCF/NEVPT2) [97,98,99], applied on the wave function that was previously obtained from complete active space (CAS) methodology, were carried out on the real nickel(II) environments in **1** and **2** to evaluate the parameters that determine the axial *zfs* (*D*). Version 4.0.1 of the ORCA program [100] through the TZVP basis set proposed by Ahlrichs [101,102] and the auxiliary TZV/C Coulomb fitting basis set was used to perform the calculations [103,104,105]. In the CASSCF procedure, the orbitals were optimized for an average of ten triplet [^3^F and ^3^P terms of the free Ni^II^ ion] and fifteen singlet (^1^G, ^1^D and ^1^S terms) roots. The contributions to *zfs* (*D*) from ten triplet and fifteen singlet excited states generated by an active space with eight electrons in five 3d orbitals were included using an effective Hamiltonian. The RIJCOSX method was applied, combining resolutions from the identity (RI) and “chain of spheres” COSX approximations for the Coulomb and exchange terms, respectively [106,107,108].

## 4. Conclusions

In conclusion, the magnetic dynamics of the mononuclear nickel(II) complexes **1** and **2**, which contain functionalized terpy derivatives as ligands, are governed by two-phonon Orbach-type relaxation, except at temperatures close to 2.0 K or lower, where a one-phonon direct mechanism seems to allow faster spin reversal. It deserves to be outlined that the number of magneto-structurally characterized SIMs of six-coordinate mononuclear Ni(II) complexes is really small. As far as we know, only three mononuclear six-coordinate nickel(II) complexes of the formulas [Ni(pydm)_2_](dnbz)_2_ [pydm = 2,6-bis(hydroxymethyl)pyridine and Hdnbz = 1.5-dinitropbenzoic acid] [84], [Ni(NCS)_2_(nqu)_2_)]∙2nqu (nqu = 5-nitroquinoline) [85] and [Ni(pydc)(pydm)]∙H_2_O (H_2_pydc = pyridine-2,6-dicarboxylic acid) [86] have been reported to exhibit slow magnetic relaxation. In the present contribution, **1** and **2** represent two rare cases of mononuclear six-coordinate nickel(II) behaving as field-induced SIMs that are added to this reduced family of compounds. Finally, the more remarkable aspect of this contribution, apart from driving fascinating properties of interest in the future development of nanodevices, lies in the availability of connectors (carboxylate or pyridine groups) that could act as donors toward other metal ions forming elaborated homo- and heterometallic systems. The new attached metal sites will afford additional chemical or physical properties, which could be coupled to those already present in **1** and **2**, leading to synergy and also opening the door toward advanced multifunctional systems. This is a research avenue that deserves to be explored in the near future.

## Figures and Tables

**Table 1:** Crystal data and structure refinement for **1** and **2**.

	1	2
Formula	C_32_H_30_Cl_2_N_6_NiO_16_	C_45_H_32_Cl_2_N_8_NiO_9_
*Fw*	884.23	958.39
Crystal	Monoclinic	Triclinic
Space	*P*21/*c*	*P*-1
*a*/Ǻ	9.422(2)	8.9014(4)
*b*/Ǻ	11.852(3)	13.8843(7)
*c*/Ǻ α/°	34.719(8) 90	18.1919(9) 91.026(2)
β/° γ/°	91.926(13) 90	94.525(2) 108.521(2)
*V*/Ǻ^3^	3874.7(17)	2123.04(18)
*Z*	4	2
*D_c_*/g cm^−3^	1.516	1.499
*T*/K	296(2)	150
μ/mm^−1^	0.651	0.651
*F*(000)	1816	984
ϴ range for data collection (°)	2.426–24.997	2.248–27.000
Index ranges	−11 ≤ *h* ≤ 11 −14 ≤ *k* ≤ 14 −41 ≤ *l* ≤ 41	−11 ≤ *h* ≤ 11 −17 ≤ *k* ≤ 17 −23 ≤ *l* ≤ 23
Refl. collected	58,849	96,423
Refinement method	Full-matrix least-squares on *F*^2^	Full-matrix least-squares on *F*^2^
Refl. independent	6791 [*R*(int) = 0.0979]	9260 [*R*(int) = 0.0337]
Data/restraints/param.	6791/7/517	9260/9/631
Goodness-of-fit on *F*^2^	1.054	1.012
Final *R* indices ^1,2^ [*I* > 2σ(*I*)]	*R*_1_ = 0.0966 *wR*_2_ = 0.2543	*R*_1_ = 0.0359 *wR*_2_ = 0.0988
*R* indices (all data)	*R*_1_ = 0.1428 *wR*_2_ = 0.2818	*R*_1_ = 0.0379 *wR*_2_ = 0.1009
∆ρ_max,min_/e Å^−3^)	0.835/−0.407	0.626/−0.692

^1^*R*_1_ = ∑(|*F*_o_|-|*F*_c_|)/∑|*F*_o_|. ^2^ *wR*_2_ = {∑[*w*(*F*_o_^2^-*F*_c_^2^)^2^]/∑[*w*(*F*_o_^2^)^2^]}^1/2^.

**Table 2:** Selected bond lengths (Å) and angles (deg) for **1**.

Ni(1)-N(1)	2.111(6)	Ni(1)-N(4)	2.112(7)
Ni(1)-N(2)	2.008(6)	Ni(1)-N(5)	2.003(6)
Ni(1)-N(3)	2.107(7)	Ni(1)-N(6)	2.116(7)
N(5)-Ni(1)-N(2)	178.7(3)	N(2)-Ni(1)-N(4)	103.3(3)
N(5)-Ni(1)-N(3)	101.6(3)	N(3)-Ni(1)-N(4)	95.5(3)
N(2)-Ni(1)-N(3)	77.6(3)	N(1)-Ni(1)-N(4)	89.6(3)
N(5)-Ni(1)-N(1)	102.8(3)	N(5)-Ni(1)-N(6)	78.2(3)
N(2)-Ni(1)-N(1)	78.1(3)	N(2)-Ni(1)-N(6)	100.8(3)
N(3)-Ni(1)-N(1)	155.6(3)	N(3)-Ni(1)-N(6)	89.8(3)
N(5)-Ni(1)-N(4)	77.7(3)	N(1)-Ni(1)-N(6)	95.2(3)
N(4)-Ni(1)-N(6)	155.9(2)		

**Table 3:** Selected bond lengths (Å) and angles (deg) for **2**.

Ni(1)-N(1)	2.1294(14)	Ni(1)-N(5)	2.1268(14)
Ni(1)-N(2)	2.0002(14)	Ni(1)-N(6)	1.9988(14)
Ni(2)-N(3)	2.1182(14)	Ni(2)-N(7)	2.1202(14)
N(6)-Ni(1)-N(2)	179.16(6)	N(3)-Ni(1)-N(5)	92.15(5)
N(6)-Ni(1)-N(3)	102.70(5)	N(7)-Ni(1)-N(5)	155.58(5)
N(2)-Ni(1)-N(3)	77.74(5)	N(6)-Ni(1)-N(1)	101.51(5)
N(6)-Ni(1)-N(7)	77.95(5)	N(2)-Ni(1)-N(1)	78.04(5)
N(2)-Ni(1)-N(7)	101.32(5)	N(3)-Ni(1)-N(1)	155.78(6)
N(3)-Ni(1)-N(7)	94.18(5)	N(7)-Ni(1)-N(1)	90.30(5)
N(6)-Ni(1)-N(5)	77.66(5)	N(5)-Ni(1)-N(1)	93.53(5)
N(2)-Ni(1)-N(5)	103.06(5)		

## Data Availability

Not applicable.

## Supplementary Materials

The following are available at https://www.mdpi.com/article/10.3390/molecules28114423/s1, Figures S1 and S2: Infrared spectra of **1** and **2**. Figures S3 and S4: XRPD patterns for **1** and **2** compared to the calculated ones. Figure S5: Thermal study of **1** and **2**. Figures S6–S9: Crystallographic drawings for **1**. Figures S10–S13: Crystallographic drawings for **2**. Figures S14: χ_M_*T* vs. *T* and *M* against *T* plot for **1**. Figure S15: Relative orientation of the experimental coordination environment and the calculated *D* tensors for **1** and **2**. Figure S16: Temperature dependence of χ_M_′ and χ_M_″ as a function of the applied dc field for **1** and **2**. Figure S17: Frequency dependence of χ_M_′ and χ_M_″ and Argand plots for **1** and **2** under applied dc fields of 2.0 and 5.0 kOe. Table S1: Hydrogen bonds for 1. Table S2: Energy of the calculated quartet and triplet excited states and their contributions to the *D* and *E* values for **1** and **2**.

## Sample Availability

Samples of the compounds are available from the authors.
