# Supplementary material for: Field-Induced Single-Ion Magnet Behavior in Nickel(II) Complexes with Functionalized 2,2′:6′-2″-Terpyridine Derivatives: Preparation and Magneto-Structural Study
Source: Molecules. 2023 May 29;28(11):4423. doi: 10.3390/molecules28114423 (PMC10254432; doi:10.3390/molecules28114423)
Supplement: Supplementary file 1 [file molecules-28-04423-s001.zip › molecules-2413879-supplementary.pdf]

Supplementary Materials

Field-Induced Single-Ion Magnet Behavior in Nickel(II) Complexes with Functionalized 2,2′:6′-2′′-Terpyridine Derivatives: Preparation and Magneto-Structural Study

Francisco Ramón Fortea-Pérez [1], Julia Vallejo [1], Teresa F. Mastropietro [2], Giovanni De Munno [2,*], Renato Rabelo [1,3], Joan Cano [1,*] and Miguel Julve [1,*]

[1] Instituto de Ciencia Molecular (ICMol), Departament de Química Inorgànica, Universitat de València,
46980 Paterna, Spain; francisco.fortea@uv.uv (F.R.F.-P.); julia.vallejo@uv.es (J.V.); renato.rabelo@uv.es (R.R.)

[2] Dipartimento di Chimica e Tecnologie Chimiche, Università della Calabria, 87036 Rende, Italy; teresafina.mastropietro@unical.it

[3] Instituto de Química, Universidade Federal de Goiás, 74690-900 Goiânia, Brazil

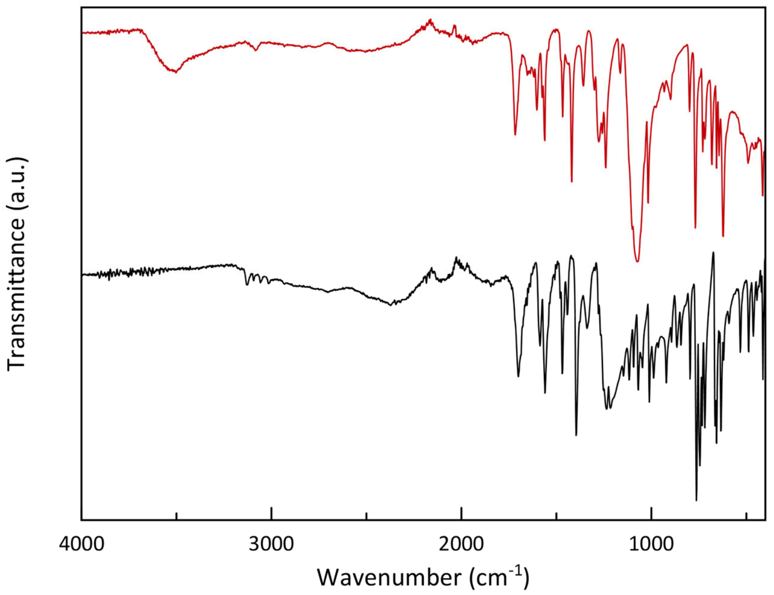

Figure S1. Infrared spectrum of **1** (red) compared to the ligand terpyCOOH (black).

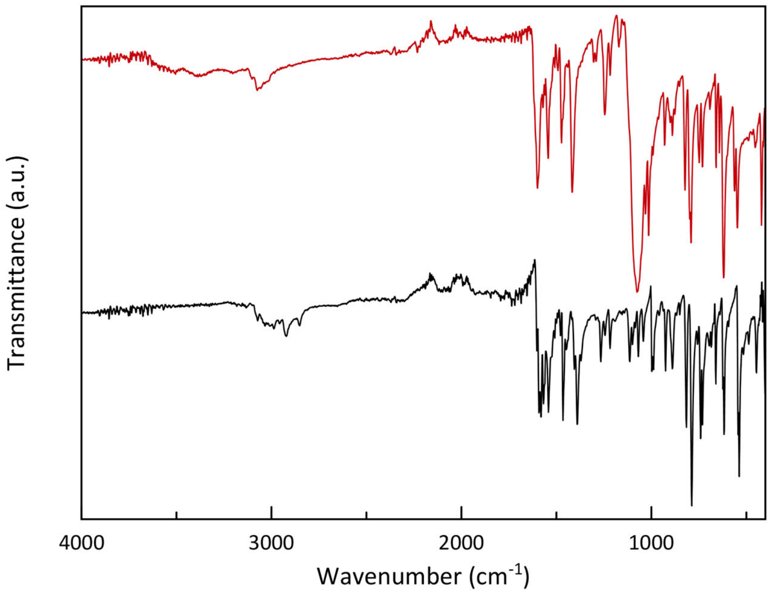

Figure S2. Infrared spectrum of **2** (red) compared to the ligand terpyepy (black).

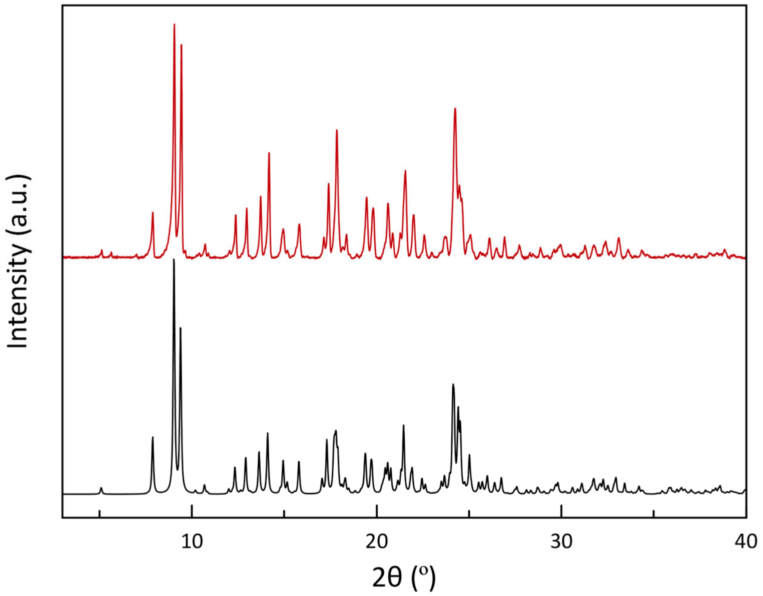

Figure S3. Calculated (black) and experimental (red) X-ray diffraction patterns for **1**.

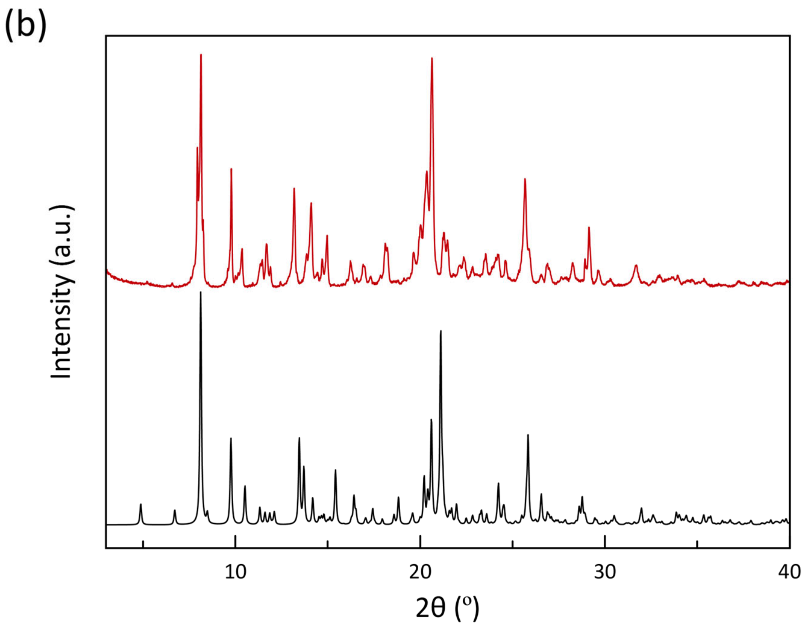

Figure S4. Calculated (black) and experimental (red) X-ray diffraction patterns for **2**.

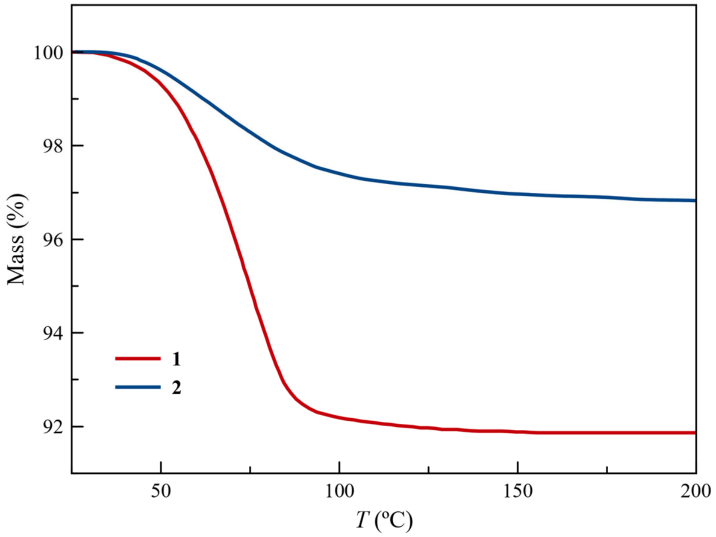

Figure S5. Thermogravimetric behavior of **1** and **2** under a dinitrogen flow of 100 mL min^{-1} with a heating rate of 10 ºC min^{-1}.

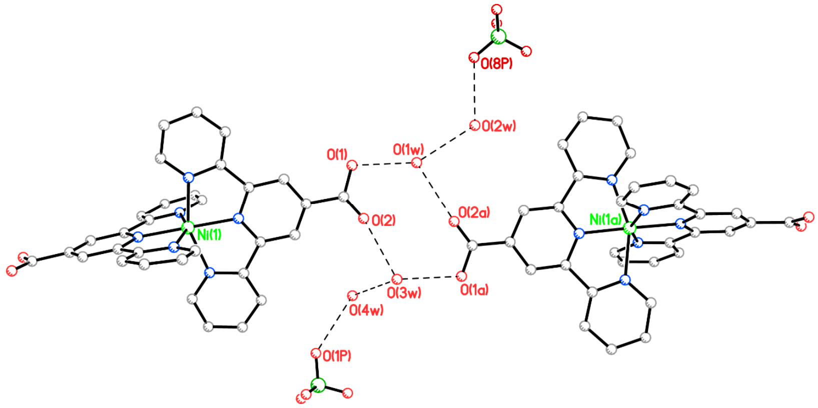

Figure S6. View of the hydrogen bonding pattern in **1** illustrating the occurrence of a supramolecular six-membered ring.

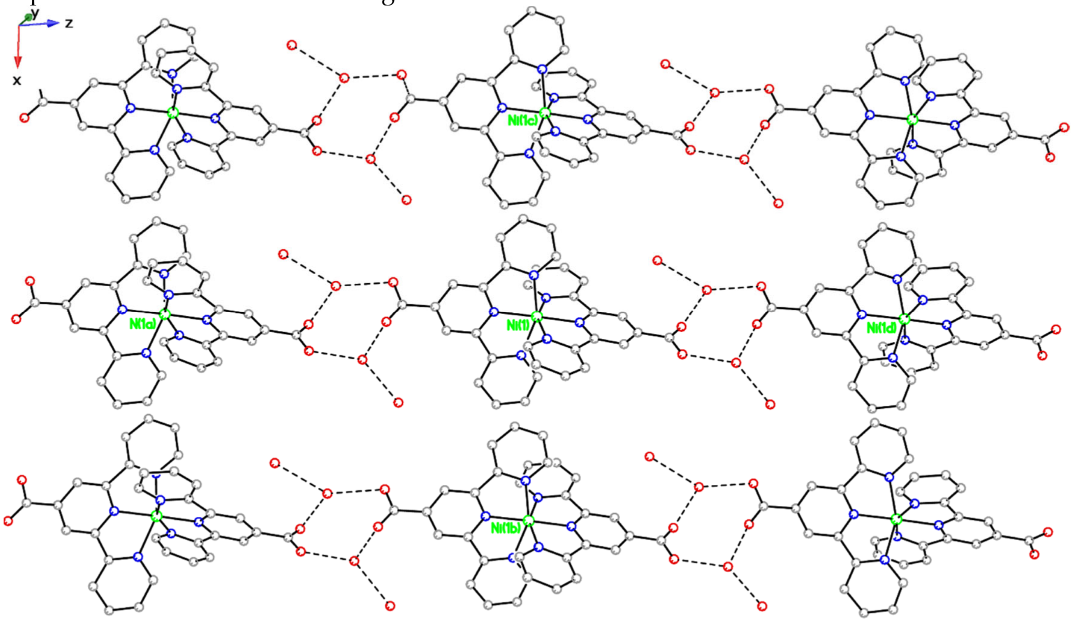

(a)

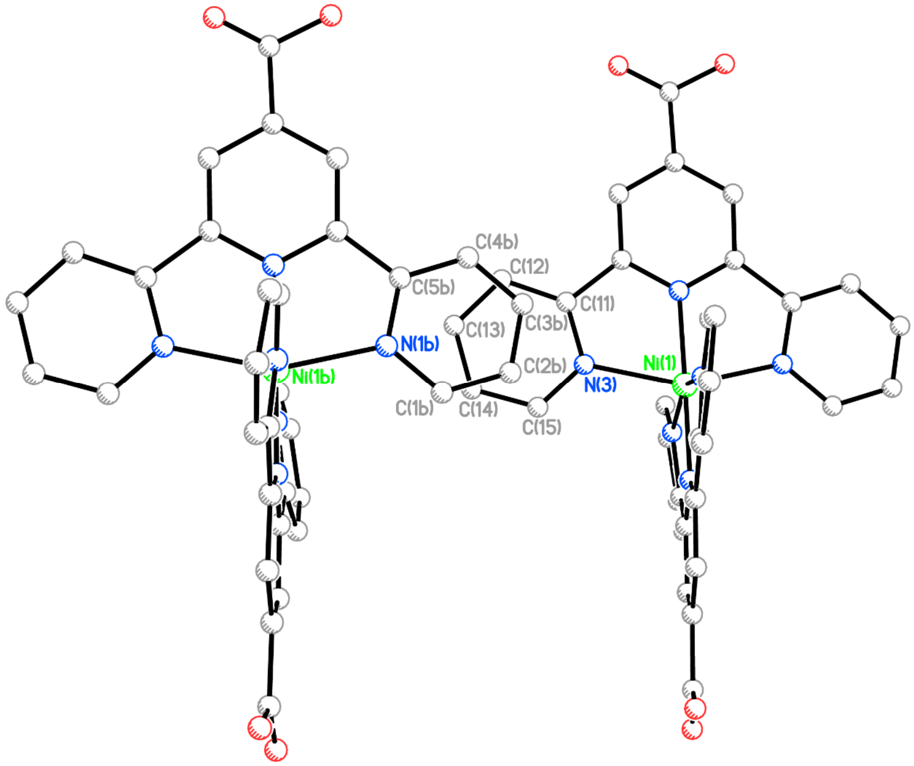

(b)

Figure S7. (a) Hydrogen bonding interactions along the crystallographic *c* axis and π-π stacking between the N(1)/N(3) pyridyl rings of one terpyCOOH ligand leading to a supramolecular 2D motif in the *ac* plane in **1**. (b) A detail of the π- π interactions between yhe outer N(1) and N(3) pyridyl rings of one ligand, which propagate along the crystallographic *a* axis [N(1)/N(3*b* and, N(3)/N(1*c*); symmetry code: (b) = 1 + *x*, *y*, *z*; *(c)* = -1 + *x*, *y*, *z*)] in **1**.

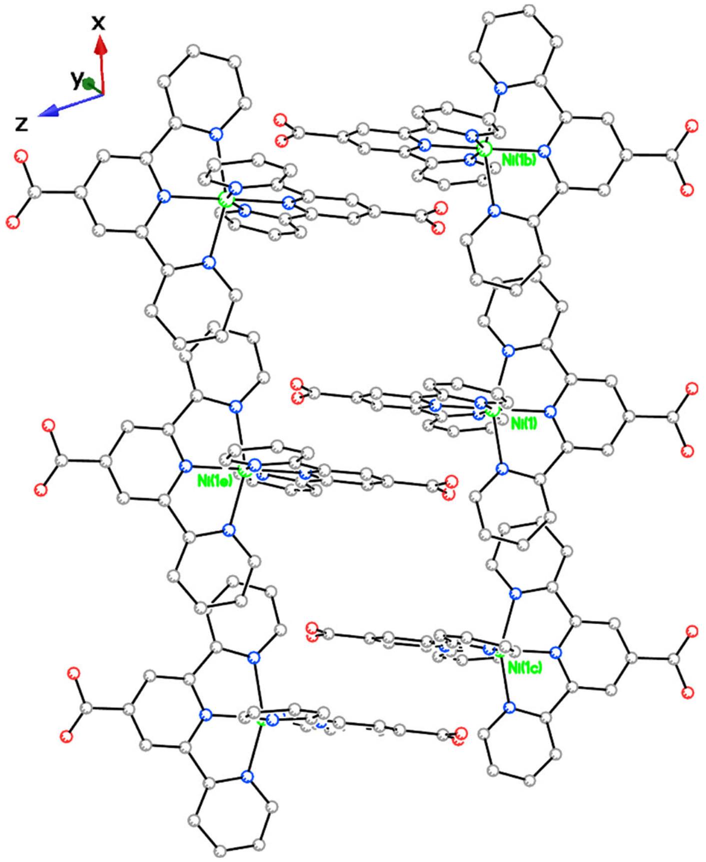

(a)

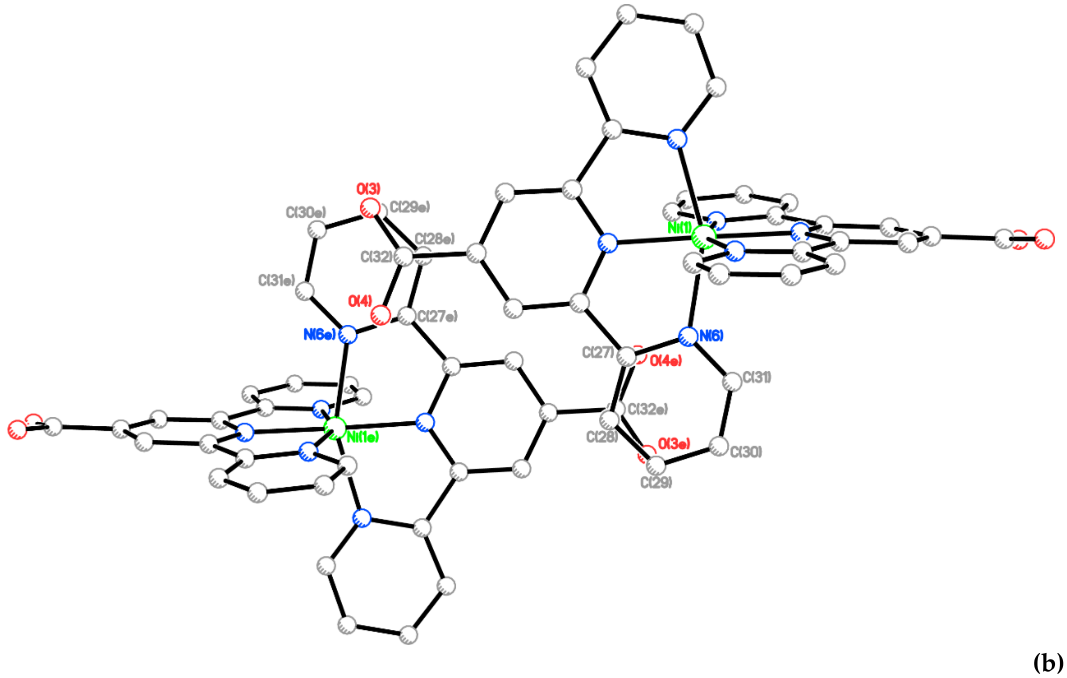

(b)

Figure S8. a) and b) Views of the stacking-like interactions between the N(6) pyridyl ring of one terpyCOOH molecule and the C(32)-O(2)-O(3) carboxylic group of an adjacent terpyCOOH ligand in **1** [symmetry code: ¡ (e) = 1 - *x*, -*y*, -*z*)].

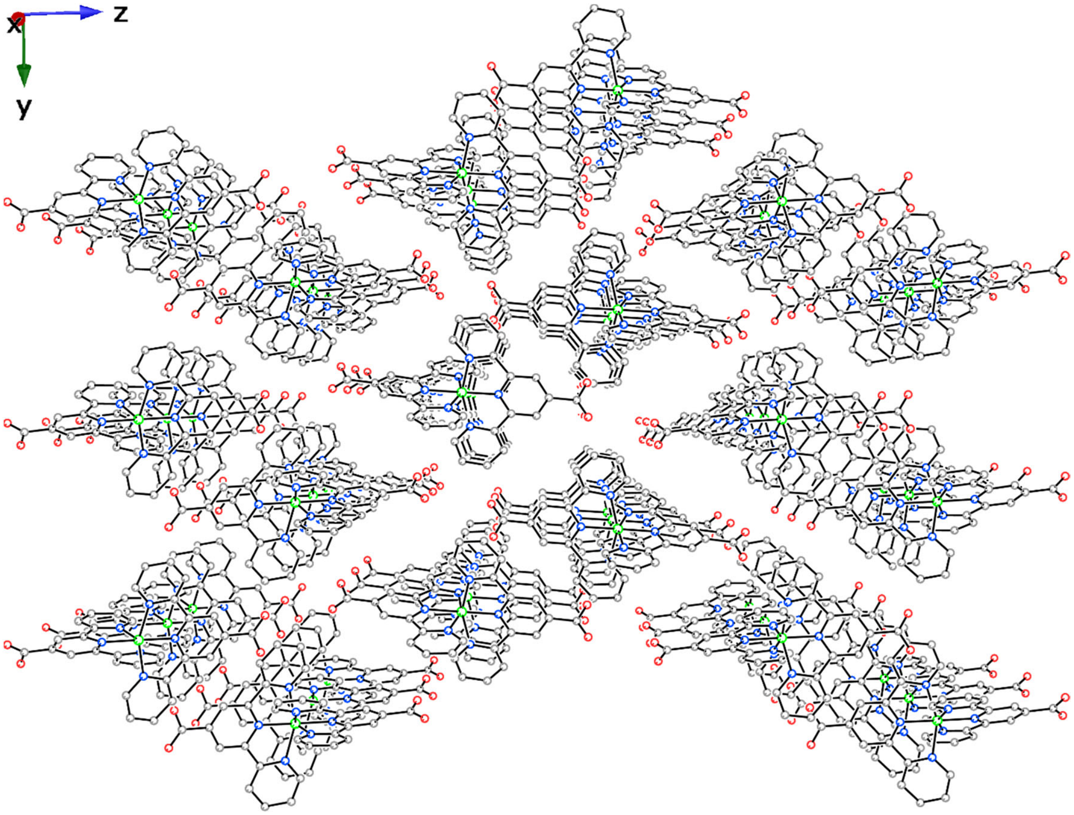

Figure S9. View of the supramolecular 3D arrangement in **1** supported by additional hydrogen bonds involving the water molecules of crystallization and the perchlorate anions.

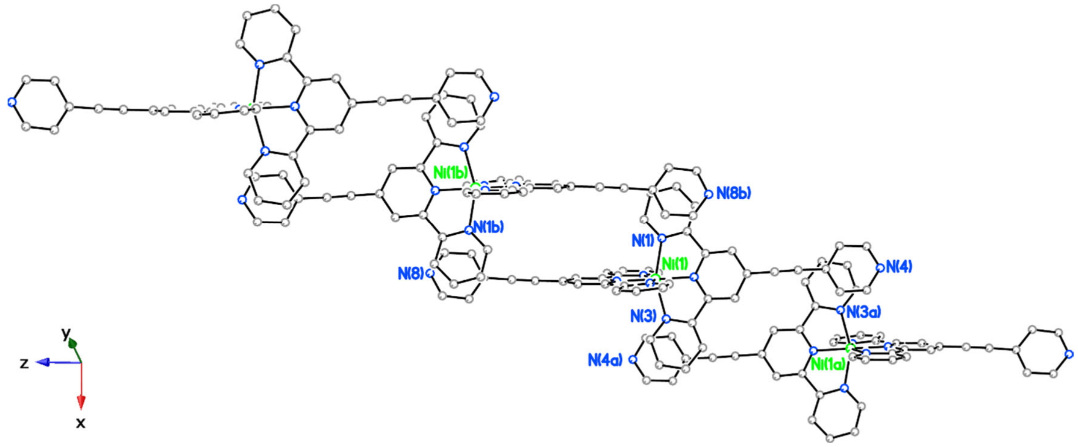

(a)

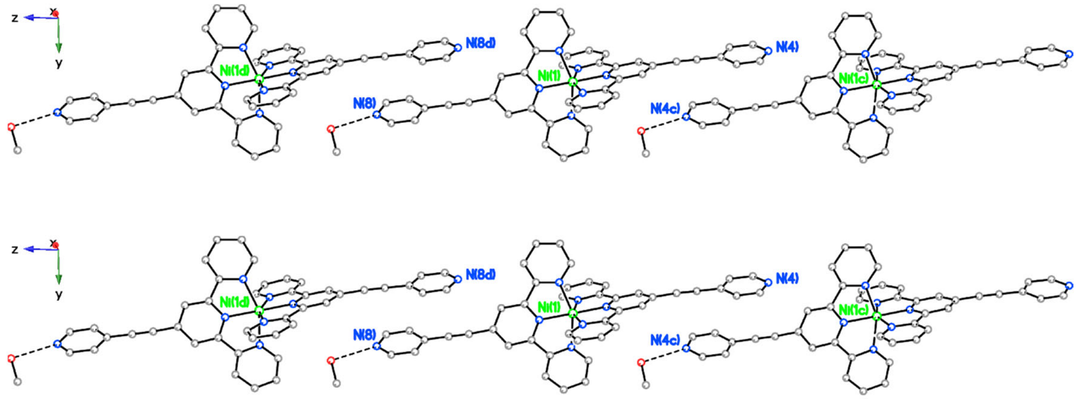

(b)

Figure S10. (a) A view of the supramolecular 1D motif in **2** growing along the *bc* diagonal being assembled by π- π interactions between the $[Ni(terpyepy)_2]^{2+}$ complex cations where the epy groups and the outer pyridyl rings of only one terpyepy ligand participate [symmetry code: *(a)* = 1 - *x*, -*y*, -*z*; (b) = 1 - *x*, 1 - *y*, 1 - *z*]. (b) Additional stacking interactions between the epy pyridyl rings containing the N(4) and N(8) atoms along the crystallographic *z* axis in **2**.

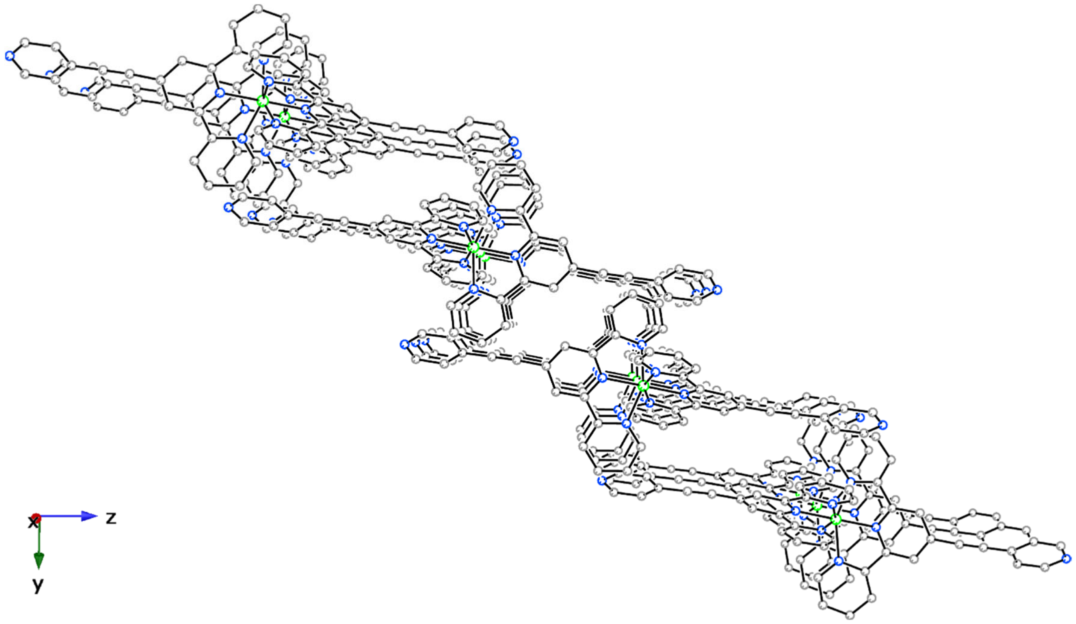

Figure S11. View of a fragment of the supramolecular 2D motif arising from π-π interactions between the $[Ni(terpyepy)_2]^{2+}$ complex cations in **2**.

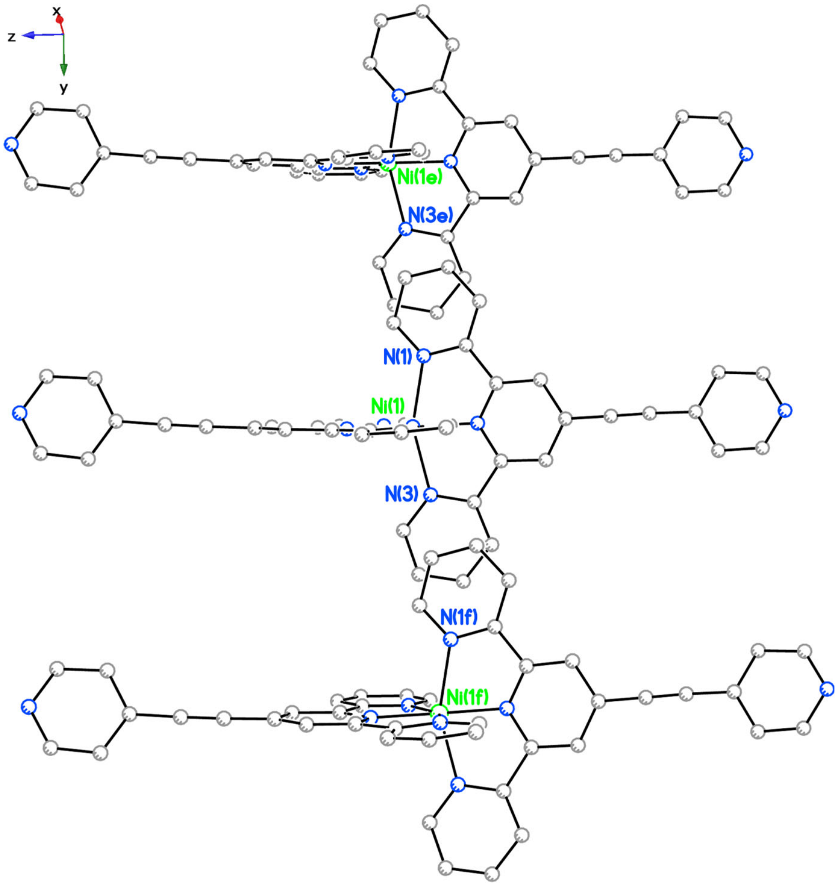

Figure S12. π- π interactions occurring between the pyridyl rings containing the N(1) and N(3*e*) and N(3) and N(1*f*) atoms [symmetry code: (e) = -1 + x, y, z; (f) = 1 + x, y, z] atoms along the crystallographic *x* axis.

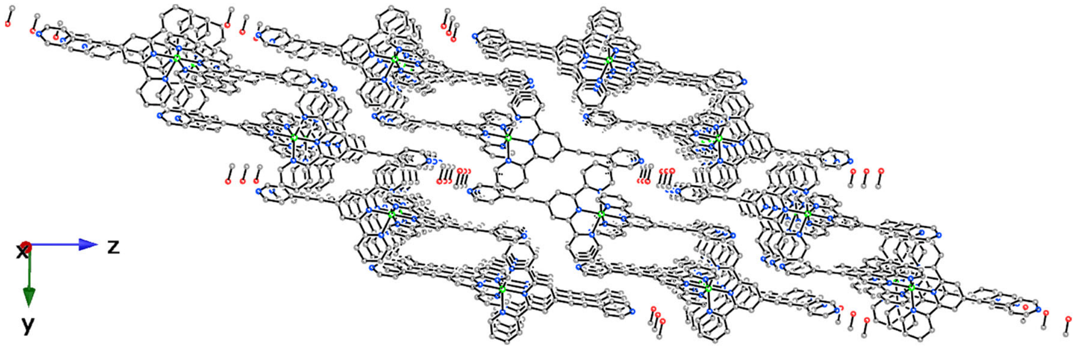

Figure S13. A view of the supramolecular 3D assembly in **2** showing the methanol solvent molecules arranged in small channels formed along thecrystallographic *a* axis.

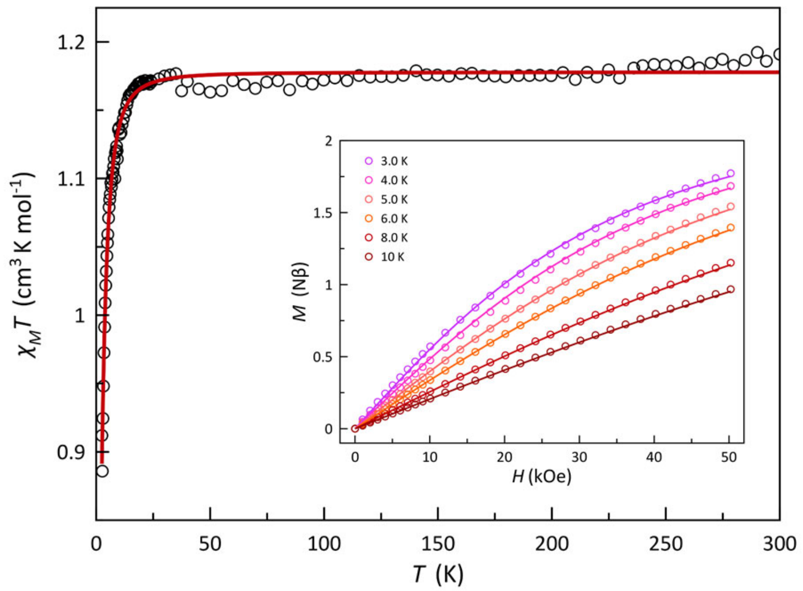

Figure S14. $\chi_M T$ vs. T and M vs. H plots (inset) for **1**: (empty circles) experimental; (solid line) best-fit curves to the experimental data using the parameters reported in the text according to the *zfs* splitting approach (see text).

(a)

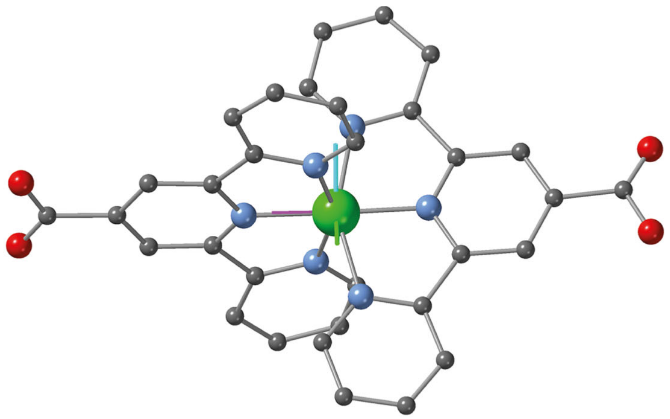

(b)

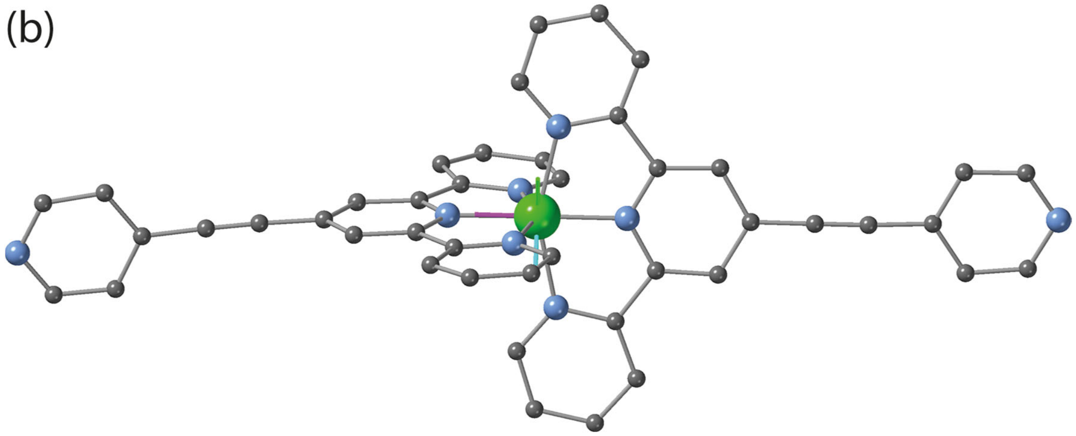

Fig. S15. Relative orientations of the experimental coordination environment and the calculated *D* tensor (*x* = cyan, *y* = green, *z* = magenta) for **1** (a) and **2** (b). Color code: green, nickel; light blue, nitrogen; red, oxygen; grey, carbon. Hydrogen atoms are omitted for clarity.

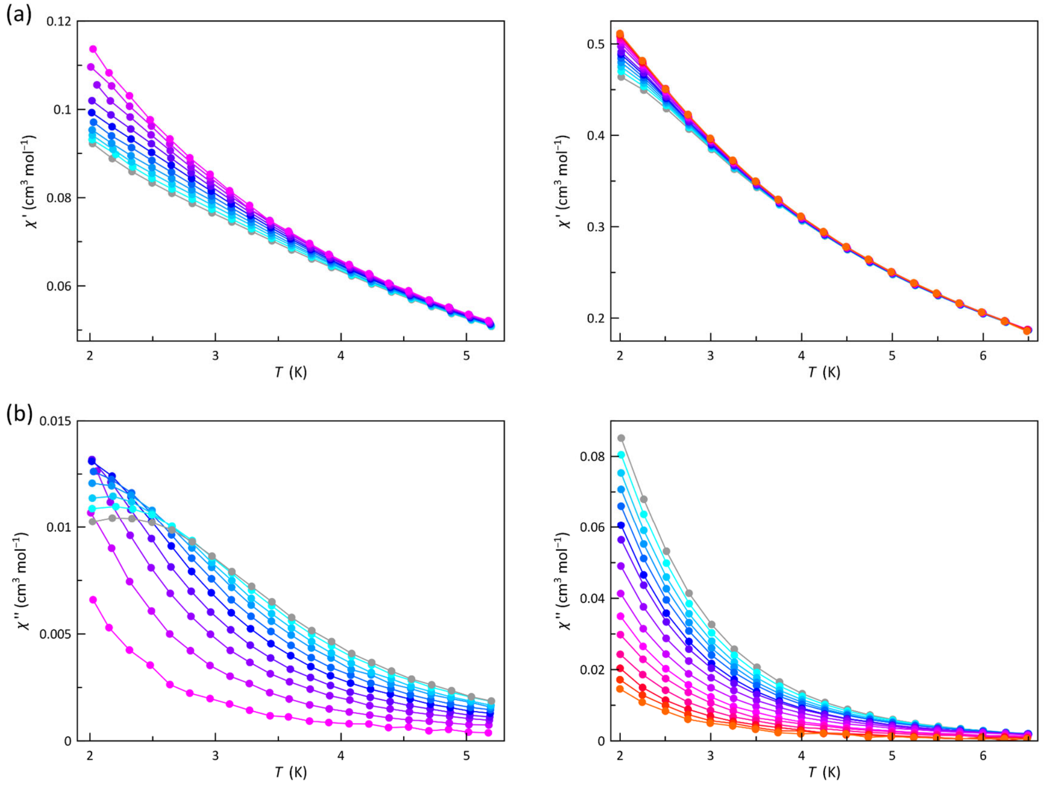

Figure S16. Temperature dependence of ◎M′ (a) and ◎M′′ (b) of **1** (left) and **2** (right) under a static magnetic field of 2.0 and 5.0 kOe, respectively, at ± 5 Oe oscillating magnetic field in the frequency range 10.0-1.0 kHz (from gray to warmer colors).

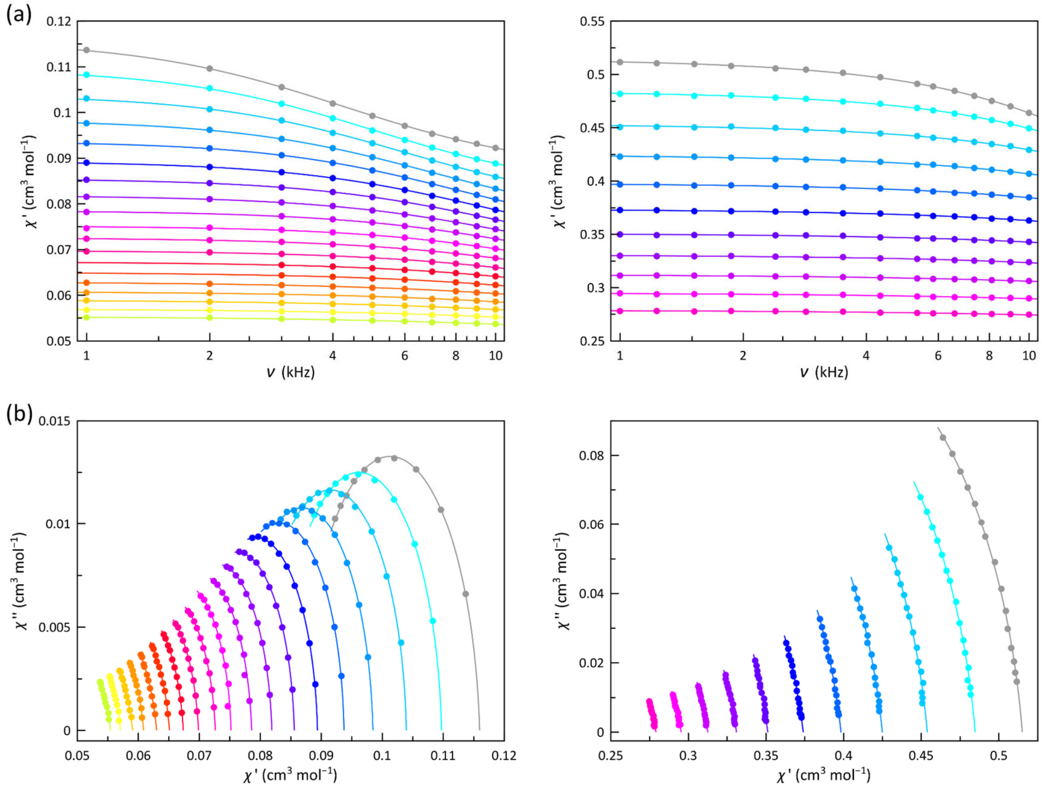

Figure S17. Frequency dependence of χ_M' (a) and the Argand plot (b) of **1** (left) and **2** (right) under a static magnetic field of 2.0 and 5.0 kOe, respectively, at ± 5 Oe oscillating magnetic field. The solid lines are the best-fit curves obtained through the generalized Debye model.

Table S1. Hydrogen bonds for **1***,&

D-H···A	d(D-H) [Å]	d(H...A) [Å]	d(D···A) [Å]	<(DHA) [º]
O(4)-H(4A)...O(3W)#1	0.82	1.78	2.598(9)	176.4
O(1)-H(1A)...O(1W)	0.82	1.78	2.600(10)	174.5
O(1W)-H(1W)...O(3)#2	0.95(3)	1.85(3)	2.803(11)	175(12)
O(1W)-H(2W)...O(2W)	0.95(3)	1.92(8)	2.769(12)	148(12)
O(3W)-H(5W)...O(4W)	0.96(3)	1.90(7)	2.795(14)	155(13)
O(3W)-H(6W)...O(2)	0.96(3)	1.87(7)	2.781(12)	158(13)
O(4W)-H(8W)...O(4P)#3	0.97(3)	2.04(8)	2.93(2)	151(13)
O(2W)-H(3W)...O(8P)#4	0.96(3)	2.39(11)	3.02(3)	123(10)

*D = donor and A = acceptor. &Symmetry transformations used to generate equivalent atoms: (#1) = x, –y + ½, z + ½; (#2) = x, –y + ½, z –½; (#3) = –x + 2, y – ½, –z + 1/2; (#4) = –x + 1, y – ½, –z + ½.

Table S2. Energy of the calculated quartet (Q_i) and triplet (D_i) excited states and their contributions to the *D* and *E* values for **1** and **2** obtained from CASSCF/NEVPT2 calculations. D_{SS} is the spin-spin contribution to axial *zfs* parameter, and D_T and D_S stand for the sum of spin-orbit contributions coming from quartet and doublet excited states

1					2				
State	Energy[a]	*S*	*D*[a]	*E*[a]	State	Energy[a]	*S*	*D*[a]	*E*[a]
D_{SS}		1	+0.035	+0.011	D_{SS}		1	–0.031	–0.008
D_T		1	–7.331	–0.330	D_T		1	–8.351	–0.323
D_S		0	+1.960	–0.043	D_S		0	+2.254	–0.011
T_1	10795.6	1	–39.758	+0.007	T_1	10429.0	1	–41.142	–0.002
T_2	13062.7	1	+16.090	–6.175	T_2	12916.7	1	+16.215	–13.440
T_3	13186.5	1	+15.939	+5.933	T_3	12974.9	1	+15.985	+13.187
T_4	20039.6	1	+0.232	–0.208	T_4	19759.1	1	+0.311	–0.309
T_5	20417.1	1	+0.142	+0.114	T_5	20006.7	1	+0.245	+0.243
T_6	21361.0	1	+0.001	+0.002	T_6	21153.7	1	+0.001	–0.001
T_7	31037.8	1	+0.010	+0.010	T_7	30681.7	1	+0.017	+0.016
T_8	31318.1	1	+0.013	–0.013	T_8	30968.9	1	+0.017	–0.017
T_9	32127.6	1	–0.000	–0.000	T_9	31962.6	1	+0.000	+0.000
S_1	15911.8	0	+0.002	–0.000	S_1	15666.9	0	+0.001	+0.000
S_2	16489.7	0	–0.000	+0.000	S_2	16470.7	0	–0.000	–0.000
S_3	27026.5	0	+14.394	–0.001	S_3	26637.7	0	+14.581	–0.000
S_4	28990.6	0	–6.201	+6.097	S_4	28732.0	0	–6.159	+6.141
S_5	29218.9	0	–6.235	–6.139	S_5	28879.9	0	–6.169	–6.152

[a]Values in cm^{-1}.
